# Implications of the gut microbiome in spinal cord injuries

**DOI:** 10.3389/fsurg.2025.1668225

**Published:** 2025-10-16

**Authors:** Naveen Jeyaraman, Madhan Jeyaraman, Priya Dhanabal, Swaminathan Ramasubramanian, Luca Ambrosio, Gianluca Vadalà, Sathish Muthu

**Affiliations:** 1 Department of Orthopaedics, ACS Medical College and Hospital, Dr MGR Educational and Research Institute, Chennai, Tamil Nadu, India; 2 Department of Orthopaedics, Orthopaedic Research Group, Coimbatore, Tamil Nadu, India; 3 Department of Orthopaedics, Brazilian Institute of Regenerative Medicine (BIRM), São Paulo, Indaiatuba, Brazil; 4 Department of Orthopaedics, Government Medical College, Omandurar Government Estate, Chennai, Tamil Nadu, India; 5 Operative Research Unit of Orthopaedic and Trauma Surgery, Fondazione Policlinico Universitario Campus Bio-Medico, Rome, Italy; 6 Laboratory for Regenerative Orthopaedics, Departmental Faculty of Medicine and Surgery, Università Campus Bio-Medico di Roma, Rome, Italy; 7 Central Research Laboratory, Meenakshi Medical College Hospital and Research Institute, Meenakshi Academy of Higher Education and Research, Chennai, India

**Keywords:** gut microbiome, spinal cord injury, neuromodulation, neuroprotection, neuroplasticity, microbiota, spine

## Abstract

Spinal cord injuries (SCIs) present complex challenges in medical treatment and rehabilitation, profoundly affecting the patient's physiological and neurological status. Emerging research on the gut microbiome has unveiled its potential role in influencing SCI outcomes and recovery. The gut microbiome undergoes significant changes following SCIs, which influence systemic inflammation and increase susceptibility to secondary complications, such as infections and chronic pain. These effects are linked to altered permeability, immune system dysregulation, and activation of the gut-brain axis, which represent promising therapeutic targets for the treatment of these conditions. Insights into the mechanisms underlying these effects were explored, highlighting the roles of microbial-derived metabolites like short-chain fatty acids, which have been shown to possess anti-inflammatory properties and support neuroprotective responses. The implications of these findings are significant, suggesting that interventions aimed at modulating the gut microbiome, such as the use of probiotics, prebiotics, and faecal microbiota transplantation, could complement existing SCI treatments and support recovery processes. This review aims to synthesise current knowledge on the interplay between the gut microbiome and SCIs, exploring how this relationship can influence immune modulation, inflammation, and neuroplasticity, thereby affecting recovery trajectories and the necessity for interdisciplinary research approaches that integrate neurology, microbiology, and nutrition to develop holistic, effective treatment strategies for SCI patients.

## 1 Introduction

Spinal cord injury (SCI) is a debilitating condition that disrupts not only motor and sensory functions but also autonomic processes, including gastrointestinal regulation. Emerging evidence highlights the gut microbiome as a critical player in modulating systemic inflammation, neuroimmune signalling, and recovery outcomes following SCI (1). The gut-brain axis, a bidirectional communication network between the central nervous system and the enteric microbiota, has gained attention for its role in maintaining homeostasis and influencing neurological health (2–9). SCI-induced changes in gut motility, such as ileus and constipation, alter microbial transit time, leading to dysbiosis characterised by the depletion of beneficial commensals and the overgrowth of opportunistic pathogens. This microbial imbalance can exacerbate inflammation and compromise the integrity of the gut barrier, further influencing neural recovery (10). These findings suggest that targeting the gut microbiota might offer new avenues for therapeutic intervention, aiming to mitigate some of the debilitating secondary effects associated with SCIs. This burgeoning field not only enhances our understanding of SCI pathophysiology but also underscores the complexity of the systemic impacts of such injuries, highlighting the potential of gut microbiome modulation in improving outcomes for SCI patients.

Despite growing interest, the interplay between gut microbiota and SCI remains underexplored, particularly in terms of therapeutic interventions (11). This review aims to consolidate current knowledge on gut microbial dynamics post-SCI, highlighting the roles of specific microorganisms, microbial-derived metabolites such as short-chain fatty acids, and the influence of external factors like diet, antibiotics, and stress. This review uniquely integrates findings from both preclinical and clinical studies to explore the bidirectional relationship between gut microbiota and SCI recovery, offering insights into microbial modulation as a potential therapeutic strategy.

## 2 Gut microbiome: an overview

The gut microbiome comprises a complex community of microorganisms, primarily bacteria, along with viruses, fungi, and protozoa, that reside in the human gastrointestinal tract. This ecosystem is predominantly colonised by bacteria from the phyla Firmicutes and Bacteroidetes, although significant diversity exists both across and within individual humans (12, 13). The composition of this microbiota is influenced by a myriad of factors, including genetics, age, diet, lifestyle, and antibiotic use. This dynamic community plays a crucial role in the digestion of food, synthesis of vitamins (such as vitamin K and some B vitamins), and the metabolism of bile acids and other substances that are essential for maintaining health (14). In the context of SCI, disruptions in gut microbial balance can influence systemic inflammation and recovery outcomes. Table 1 summarises key gut microorganisms and their specific contributions to host physiology.

**Table 1:** Key gut microorganisms and their functional roles.

Microorganism	Classification	Functional role	Relevance to SCI
*Lactobacillus rhamnosus* (15)	Commensal	Produces lactic acid; supports gut barrier	Anti-inflammatory effects; promotes neuroprotection
*Bifidobacterium longum* (16)	Commensal	SCFA production; immune modulation	Enhances gut-brain axis signalling
*Escherichia coli* (17)	Opportunistic	Vitamin K synthesis; can become pathogenic	Overgrowth linked to inflammation post-SCI
*Clostridium difficile* (16)	Pathogenic	Toxin production; disrupts gut flora	Risk increases with antibiotic use in SCI patients
*Akkermansia muciniphila* (18)	Commensal	Mucin degradation; maintains gut lining	Associated with metabolic health and immune balance

**Figure 1:**
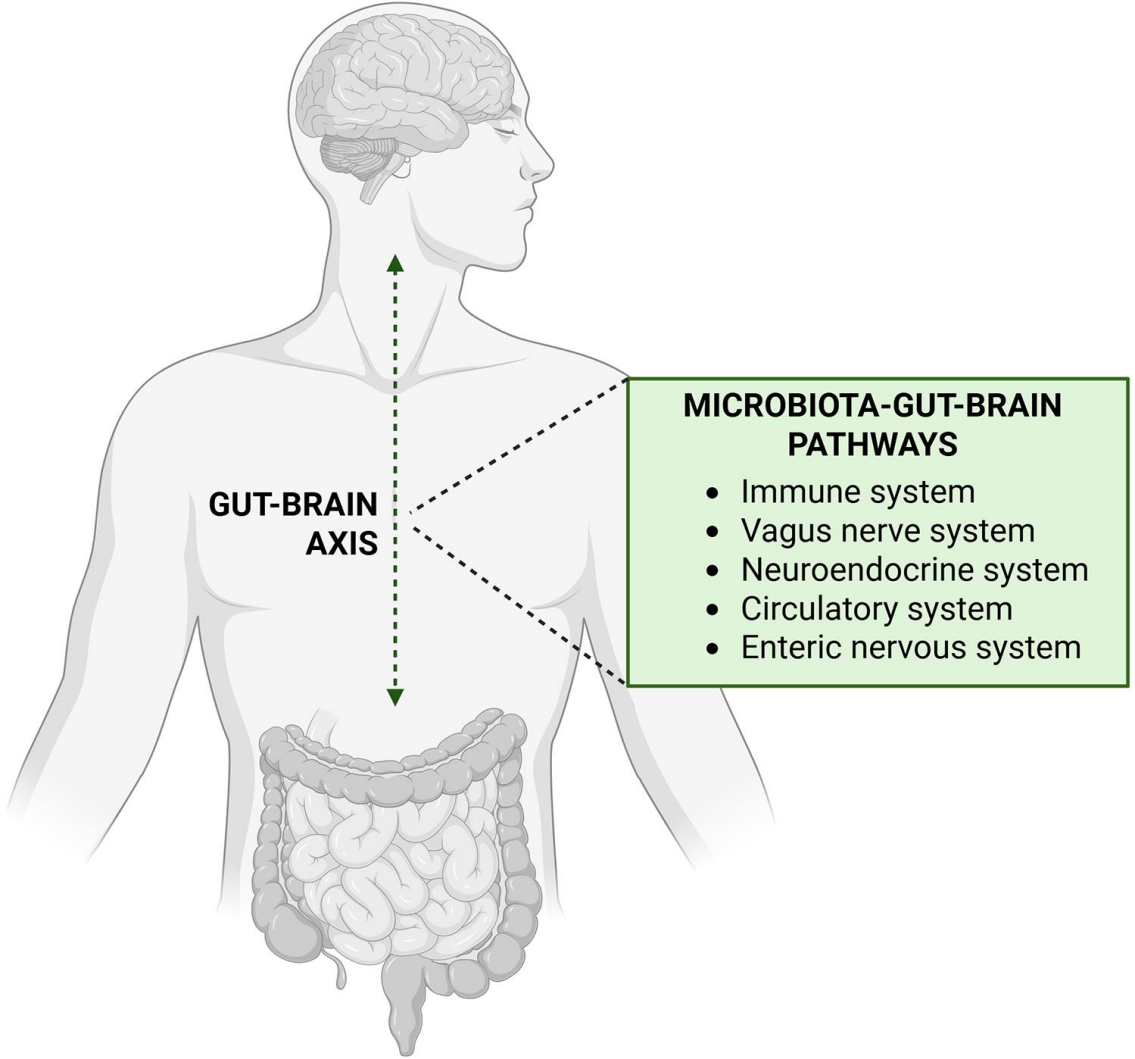
Pathways of communication in gut-brain axis. Created with http://www.BioRender.com.

The human microbiome, particularly the gut microbiome, plays a pivotal role in maintaining physiological homeostasis, serving as a critical interface between the external environment and the host's metabolic and immune systems (19–21). The gut microbiota assists in digesting food that the stomach and small intestine have not fully processed, extracting nutrients and energy that are essential for human health. Additionally, it synthesises key nutrients and metabolites, such as short-chain fatty acids (SCFA), which provide energy to host cells and regulate immune responses. Recent research highlighted how these microbial products can influence metabolic health, including impacts on obesity, type 2 diabetes, and other metabolic disorders (3, 22). Moreover, the gut microbiome plays an essential role in the development and maintenance of the mucosal barrier, protecting against pathogens and maintaining immune tolerance to beneficial commensal bacteria (23). The influence of the microbiome extends beyond metabolism and immunity to affect neurobehavioral patterns, with significant implications for mental health and behaviour. The gut-brain axis—a bidirectional communication system between the central nervous system and the gut microbiota—mediates these effects through neural, hormonal, and immunological pathways as shown in Figure 1.

For instance, certain gut bacteria can produce neurotransmitters, such as serotonin and gamma-aminobutyric acid (GABA), which can affect mood and behaviour. Furthermore, it has also been demonstrated how alterations in the gut microbiota composition could influence this axis, potentially leading to or exacerbating conditions such as depression and anxiety (2). Emerging research suggests that the microbiome may also play a role in neurological conditions such as autism and Alzheimer's disease, positing it as a potential target for therapeutic interventions. These studies underline the profound impact of the gut microbiome on the host, influencing an array of biological systems and processes that contribute to overall health and disease.

The study of the gut microbiome is multifaceted, employing various techniques that range from traditional culture-based methods to advanced genomic and bioinformatics approaches. Metagenomic sequencing has revolutionised our understanding by enabling the comprehensive analysis of microbial communities without the need for culturing them in the laboratory, providing insights into the genetic potential and functional capabilities of the microbiome (24). Additionally, metabolomics and proteomics have allowed researchers to explore the metabolic interactions between these microorganisms and their host, shedding light on how these interactions impact health and disease. These studies are increasingly aided by the development of sophisticated bioinformatic tools that manage the vast amounts of data generated, enhancing our understanding of microbial ecology and its implications for human health (25).

External factors play a significant role in shaping the composition and function of the gut microbiome, with profound implications for health and disease. Diet is a major influencer, as it provides the substrates for microbial metabolism and can dramatically alter the microbial community structure (26). High-fibre diets, for instance, promote the growth of bacteria that ferment fibre into SCFAs, which are beneficial for gut health due to their anti-inflammatory properties (27). Conversely, a diet high in saturated fats and processed food can reduce microbial diversity and increase the abundance of bacteria linked to inflammatory conditions. Such dietary patterns can predispose individuals to disorders such as obesity, diabetes, and inflammatory bowel disease by modulating the gut microbial composition (28).

Similarly, the use of antibiotics has a well-documented impact on the gut microbiome, often causing a decrease in microbial diversity and the depletion of beneficial bacteria (29, 30). This disruption can lead to antibiotic-associated diarrhoea and an increased susceptibility to infections sustained by microorganisms such as *Clostridium difficile*. Chronic or repeated antibiotic exposure can lead to long-lasting changes in the gut flora, potentially contributing to chronic health issues such as asthma and obesity. Stress is another critical factor that affects the microbiome; it can alter gut motility and secretion, microbial composition, and barrier function, thereby influencing the immune response and overall health. Tomasello et al. demonstrated how psychological stress impacts the gut microbiota, potentially exacerbating conditions like irritable bowel syndrome and impacting mental health through the gut-brain axis (31). These examples underscore the intricate relationships between external factors and the gut microbiome, emphasising the need for strategies to manage these influences to maintain or restore health.

## 3 Spinal cord injuries: impact on the gut microbiome

**Figure 2:**
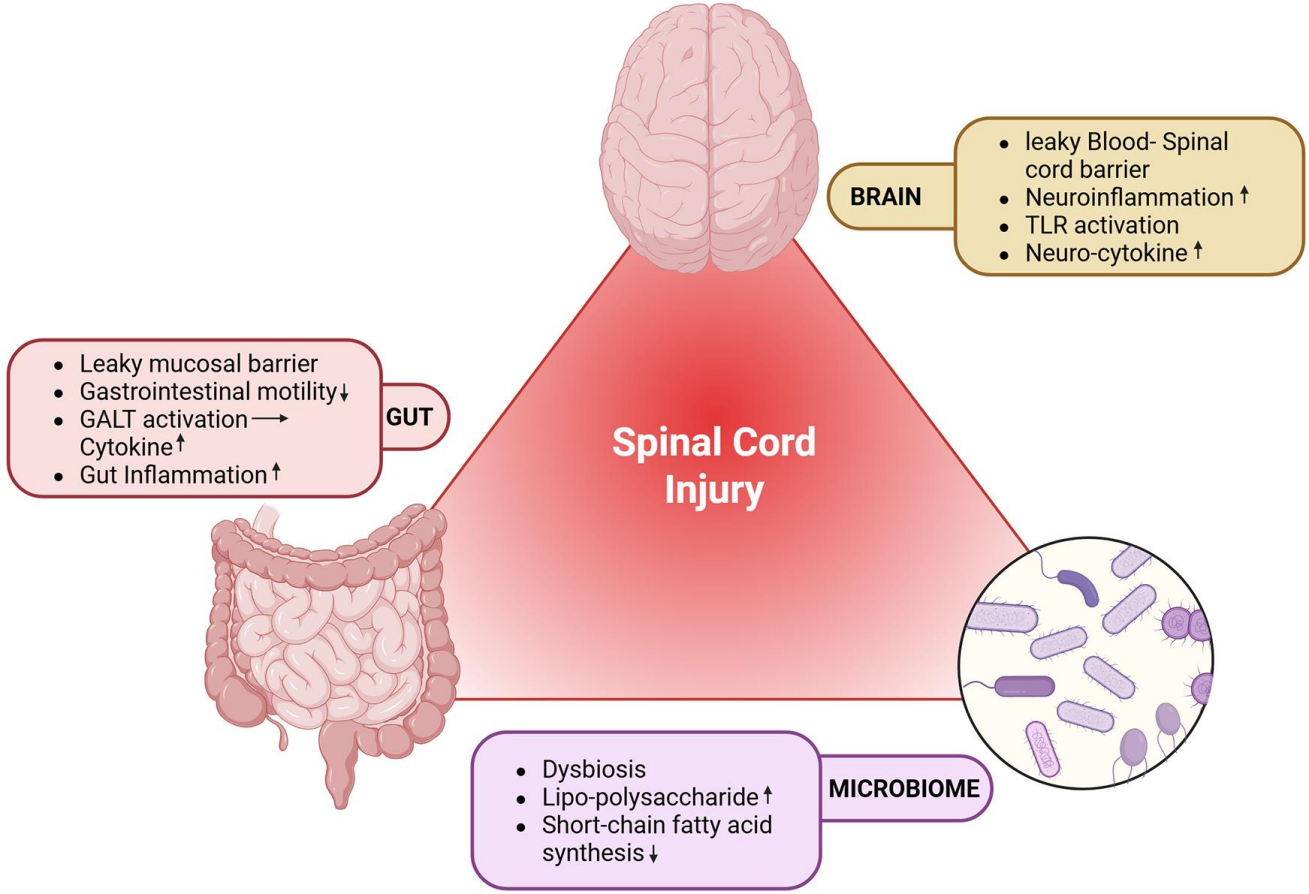
Impact of SCI on the gut microbiome. GALT, gut-associated lymphoid tissue; SCI, spinal cord injury; TLR, toll-like receptor. Created with http://www.BioRender.com.

Spinal cord injuries have profound and lasting effects on gut motility, barrier function, and the immune response, which in turn can significantly alter the diversity and composition of the gut microbiome (32). Immediately following SCI, a reduction in gut motility is common, often leading to complications such as ileus and constipation (33). This decreased motility can affect the microbial transit time, favouring the overgrowth of pathogenic bacteria at the expense of beneficial commensal populations. Over time, these changes can compromise the intestinal barrier function, enhancing the permeability of the gut barrier and potentially leading to systemic inflammation as microbial products more readily enter the bloodstream. Moreover, the immune dysregulation triggered by SCI further impacts the gut environment, as the altered immune response can change the balance of microbial populations, promoting dysbiosis (34). These alterations in microbial diversity and composition following SCI are linked to both local gut health issues and broader systemic effects, highlighting the interconnectedness of neurological injury and gut microbiome health as shown in Figure 2 (11).

Chronic inflammation is another significant concern following SCI, closely linked to alterations in the gut microbiome. The loss of the normal microbial flora and the overrepresentation of pro-inflammatory species can lead to an imbalanced immune response, characterised by systemic inflammation. This chronic inflammatory state is not only detrimental to physical health, promoting further degeneration around the injury site, but it also impacts overall recovery and rehabilitation outcomes. The inflammatory markers that surge after SCI are influenced by gut microbial products, which can permeate weakened gut barriers and enter the systemic circulation, thereby maintaining a state of chronic inflammation (35).

Neuropathic pain is a frequent and debilitating consequence of SCIs that can be exacerbated by gut microbiome alterations. The gut-brain axis, a critical communication pathway involving nervous, hormonal, and immunological signalling, is perturbed in individuals with SCI, affecting neuroinflammatory processes that can contribute to pain perception. It has also been demonstrated that specific microbial changes in the gut can influence the levels of neurotransmitters and inflammatory mediators that are known to play roles in neuropathic pain pathways. These interactions suggest that modulating the gut microbiome could be a novel approach to managing neuropathic pain in SCI patients (36). The significant changes as noted in the preclinical and clinical studies on the changes in the microbial flora following SCI is detailed in Table 2 (15, 18, 37–40).

**Table 2:** Gut microbial alterations following SCI.

Microbial group	Change after SCI	Implications
*Lactobacillus* spp.	↓ Decreased	Reduced SCFA production, impaired mucosal immunity
*Bifidobacterium* spp.	↓ Decreased	Loss of anti-inflammatory effects, compromised gut barrier
*Clostridium* spp.	↓ Decreased	Lower butyrate levels, impaired epithelial health
*Enterococcus* spp.	↑ Increased	Potential pathogenic overgrowth, risk of bacteremia
*Escherichia coli*	↑ Increased	Opportunistic infection, endotoxin release
*Proteobacteria (Klebsiella)*	↑ Increased	Dysbiosis marker, inflammation, antibiotic resistance
*Firmicutes/Bacteroidetes ratio*	Altered (↑ or ↓)	Indicator of dysbiosis, metabolic imbalance
*Akkermansia muciniphila*	↓ Decreased	Reduced mucin degradation, impaired gut barrier

The gut microbiome's role in modulating immune function after SCI extends into areas like autoimmune responses and susceptibility to systemic infections. It has also been indicated that post-SCI, shifts in gut bacterial populations can lead to increased levels of gut-derived toxins in the bloodstream, which may trigger autoimmune responses and exacerbate inflammation across the body. This phenomenon underlines the potential for targeting microbial populations to modulate immune responses and improve outcomes in SCI patients (41, 42).

Patients with SCI are frequently exposed to broad-spectrum antibiotics during acute hospitalization and rehabilitation, primarily for managing urinary tract infections, pneumonia, and pressure ulcers (43). While these interventions are clinically warranted, they inadvertently disrupt gut microbial homeostasis (44, 45). Antibiotic-induced dysbiosis leads to a marked reduction in beneficial commensals such as *Lactobacillus* and *Bifidobacterium*, impairing mucosal immunity and short-chain fatty acid production (17, 46). Concurrently, opportunistic pathogens like *Enterococcus faecalis*, *Klebsiella pneumoniae*, and *Clostridioides difficile* may proliferate, increasing the risk of systemic infections and inflammation (47). The hospital environment further exacerbates this imbalance through nosocomial microbial exposure, limited dietary fiber intake, psychological stress, and reduced mobility thereby all of which impair gut motility and barrier integrity. These factors collectively delay microbial recovery and contribute to persistent dysbiosis in SCI patients, even months post-discharge (45, 47). Recognizing these vulnerabilities underscores the need for microbiome-preserving strategies, including antibiotic stewardship, early probiotic supplementation, and dietary interventions tailored to restore microbial diversity (48). Future research should explore longitudinal microbiome dynamics in SCI cohorts and evaluate targeted therapies that mitigate hospital-acquired dysbiosis while enhancing neuroimmune resilience.

In addition to its impact on immunity, the gut microbiome after SCI affects metabolic health, influencing energy balance and glucose metabolism. These disruptions in the gut microbiota following SCI lead to impaired glucose tolerance and increased adiposity, suggesting a link between gut health and metabolic disorders in SCI patients. This emphasises the importance of maintaining a healthy microbiota to prevent the onset of metabolic disorders following neurological injury (49).

The connections between the gut microbiome and SCI-associated pathologies underscore the potential for microbiome-targeted therapies to mitigate some of the most challenging outcomes of SCIs. By restoring or modifying the gut microbiota, it may be possible to reduce infection rates, dampen chronic inflammation, and alleviate neuropathic pain, ultimately improving quality of life for individuals with SCIs. These insights into the complex interplay between the gut microbiome and various aspects of health in SCI patients highlight the potential for microbiome-based therapies to address not just local but systemic challenges posed by SCIs. As the field advances, these microbial connections offer promising avenues for comprehensive interventions aimed at improving the overall quality of life for individuals suffering from SCI.

## 4 Gut microbiome: influence on SCI recovery and complications

**Figure 3:**
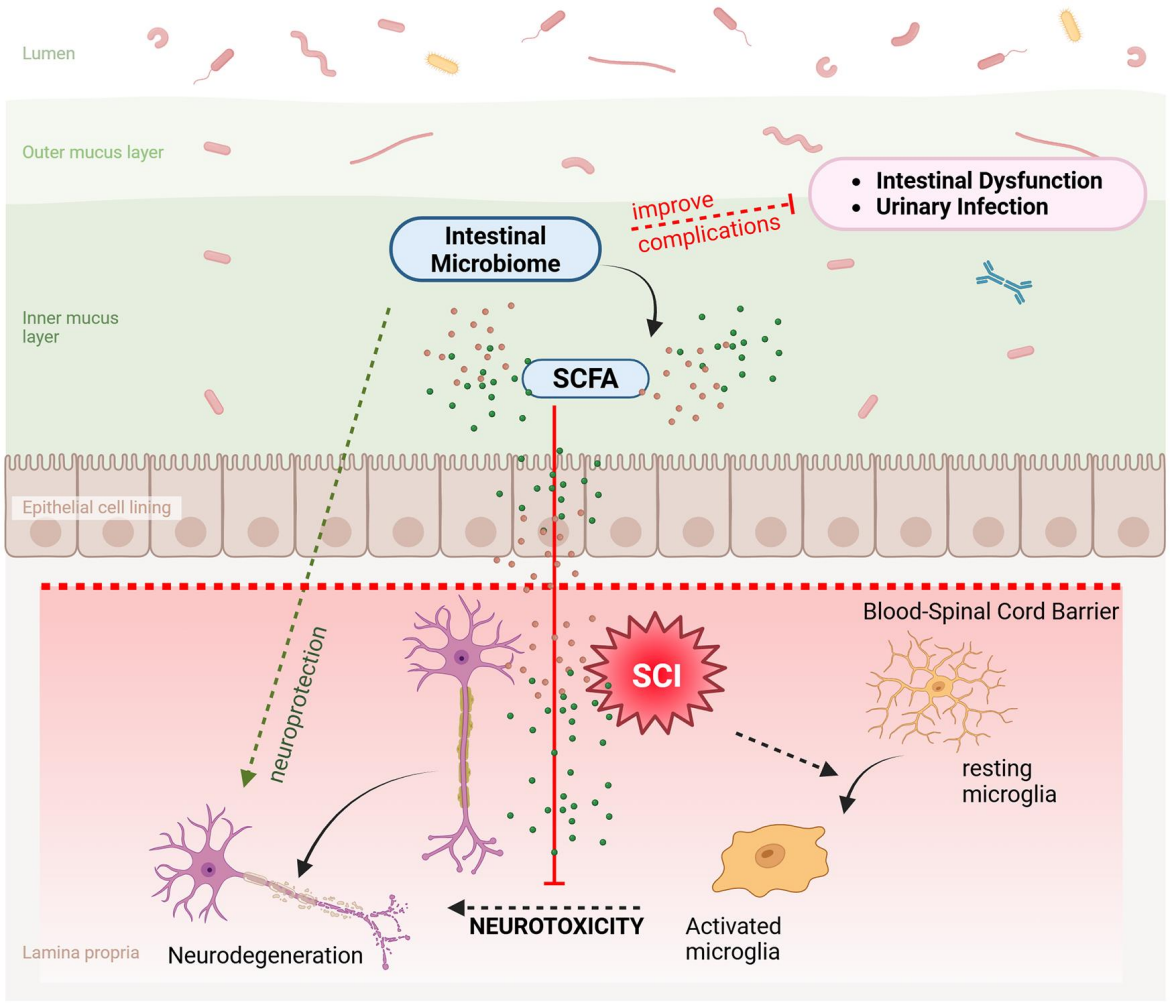
Role of the gut microbiome in SCI recovery. The intestinal microbiome secretes SCFA which inhibits the neurotoxicity caused by the activated microglia thereby resulting in neuroprotection. SCFA, short chain fatty acids; SCI, spinal cord injury. Created with http://www.BioRender.com.

The gut microbiome plays a pivotal role in influencing the recovery processes and the development of secondary complications following SCI. Research has increasingly shown that the microbiome impacts not just gut health but also systemic inflammation and immune modulation, which are crucial in the recovery phase of SCI. The microbiota can influence the healing process by affecting the immune system's response to injury and the body's ability to repair nerve damage. Microbial-derived metabolites, such as SCFAs, can facilitate neuroprotection and promote neurogenesis, which are vital for recovery after SCI. These metabolites help in modulating the immune response, reducing inflammation, and potentially aiding in the recovery of neural functions, as shown in Figure 3 (50).

Moreover, the alteration in the gut microbiome composition following SCI can lead to significant secondary complications, such as increased susceptibility to infections, gastrointestinal disturbances, and chronic pain. The disruption of the normal gut flora and the subsequent increase in pathogenic bacteria contribute to a heightened state of inflammation, exacerbating these complications. A compromised microbiome post-SCI can lead to an impaired mucosal barrier, enhancing pathogen translocation and systemic infections, which are common in individuals with severe SCIs. Previous research suggests that interventions aimed at restoring the balance of the gut microbiome could potentially mitigate these adverse effects and improve overall outcomes for SCI patients (51). These insights underline the importance of maintaining microbiome health as part of the comprehensive management and rehabilitation strategy post-SCI.

Emerging evidence suggests that specific microbial patterns within the gut can significantly influence inflammation, neuroprotection, and regenerative processes following SCIs, acting as a potential therapeutic modality (52). The diversity and composition of the gut microbiota have been linked to varying levels of inflammatory cytokines in the body, which can either exacerbate or alleviate the inflammatory response crucial in the aftermath of SCI. It has been observed that certain probiotic strains can reduce systemic inflammation by modifying the gut microbiota composition, thus potentially aiding in the recovery and repair processes post-SCI. This study illustrates how targeted probiotic supplementation can help modulate the immune response favorably during the critical phases of SCI recovery (53, 54).

SCI disrupts autonomic regulation of gastrointestinal motility, often resulting in ileus, constipation, and altered microbial transit (55). These effects are compounded by ageing, dietary insufficiencies, antibiotic exposure, and psychological stress. Ageing reduces enteric neuronal density and slows peristalsis, while low-fibre diets impair microbial fermentation and SCFA production. Antibiotics deplete commensals and promote resistant pathogens, and stress-induced sympathetic activation further delays gut transit. Collectively, these factors foster dysbiosis, marked by reduced microbial diversity, pathogenic overgrowth, and loss of beneficial taxa such as *Faecalibacterium* and *Akkermansia* (33, 56). Importantly, microbial-derived metabolites like SCFAs, tryptophan catabolites, and bile acid derivatives mediate bidirectional crosstalk with neural and immune pathways. SCFAs enhance epithelial barrier integrity, modulate microglial activation, and suppress pro-inflammatory cytokines via G-protein coupled receptors and histone deacetylase inhibition (33). This multilevel signalling promotes neuroprotection, immune tolerance, and functional recovery. A systems-level understanding of these interactions may inform targeted interventions such as prebiotic-rich diets, probiotic supplementation, and microbiome-guided rehabilitation to optimize outcomes in SCI care.

Dietary intake plays a pivotal role in shaping gut microbial composition and function, particularly in individuals recovering from SCI. Diets rich in fibre, resistant starches, and polyphenols promote the growth of beneficial taxa such as *Bifidobacterium* and *Faecalibacterium*, which in turn produce SCFAs like acetate, propionate, and butyrate. These metabolites exert anti-inflammatory effects, enhance epithelial barrier integrity, and modulate neuroimmune signalling factors crucial for SCI recovery (55). Butyrate, in particular, has been shown to support microglial homeostasis and reduce neuroinflammation via histone deacetylase inhibition. Conversely, high-fat, low-fibre diets common in institutional settings may favour dysbiotic profiles, increasing the abundance of pro-inflammatory microbes and reducing SCFA output. Emerging evidence suggests that targeted dietary interventions—such as Mediterranean-style or plant-based regimens—can restore microbial diversity and enhance SCFA-mediated neuroprotection (57). Personalised nutrition strategies, informed by microbiome profiling, may offer a promising adjunct to conventional rehabilitation by optimising microbial metabolite production and promoting systemic resilience. Future studies should explore the longitudinal impact of diet-microbiome interactions on functional outcomes in SCI populations.

Furthermore, faecal microbiota transplantation (FMT) is another therapeutic intervention receiving attention for its potential to restore a healthy gut microbiota and improve outcomes after SCI. FMT involves the transfer of faecal bacteria from a healthy donor to the gastrointestinal tract of a patient, aiming to recolonise the gut with beneficial microbes. FMT could significantly enhance neuroprotective responses and facilitate neuronal regeneration in SCI models. This process helps in establishing a more favourable microbial environment that supports the reduction of neuroinflammation and promotes recovery (58).

In addition to influencing inflammation and neuroprotection, certain microbiota compositions can also promote regenerative processes. The role of the gut-brain axis in mediating these effects is critical, as neurotransmitters and other neuroactive compounds produced by gut bacteria can influence neurogenesis and neuronal function. It has also been found that specific bacterial byproducts could activate signalling pathways that are vital for neural tissue regeneration and plasticity post-SCI (59). By manipulating the gut microbiota through dietary interventions or probiotic supplementation, it may be possible to enhance these regenerative processes and improve functional recovery. These interventions not only aim to restore a balanced gut microbiome but also leverage the gut-brain axis to mitigate adverse inflammatory responses, support neuroprotective mechanisms, and enhance regenerative processes. As the field advances, further research will likely provide deeper insights into specific microbial patterns and therapeutic strategies that can be optimised for better outcomes in SCI recovery.

Urinary tract infections (UTIs) are among the most common complications experienced by individuals with SCIs, often due to the use of catheters and the disruption of normal bladder function. The gut and urinary microbiomes are intrinsically connected, and dysbiosis in the gut can lead to an increased risk of pathogenic bacteria colonising the urinary tract. Studies have shown that probiotic supplementation can help in maintaining a balanced microbial environment, potentially reducing the incidence of UTIs in SCI patients by outcompeting pathogenic bacteria and enhancing the immune response. Targeted probiotic strains could significantly reduce the recurrence of UTIs in individuals with SCIs, suggesting that manipulating the microbiome might be an effective strategy in managing this complication (60).

Pressure ulcers are another severe complication for patients with SCI, often exacerbated by immobility and sensory loss, which leads to skin breakdown and infection (61). The skin microbiome plays a crucial role in maintaining skin integrity and barrier function, and alterations in skin flora can increase susceptibility to infections. Modulating the skin microbiome through the application of topical probiotics or microbiota-friendly skincare products can help enhance skin health and reduce the risk of pressure ulcers. It has been explored that application of topical probiotics on skin ulcer sites in SCI patients, finding that these interventions helped reduce the bacterial load and promoted faster healing, underscoring the potential of microbiota-targeted therapies in improving skin health and recovery (62).

## 5 Discussion

The field of research focusing on the gut microbiome and SCIs is burgeoning with potential but faces several significant limitations that can impact the reliability and generalizability of findings. One primary challenge is the inherent variability in study designs, which includes differences in the timing of sample collection, the methods used for characterising the microbiome, and the criteria for selecting participants. These variations can lead to inconsistent results and make it difficult to compare studies directly. For instance, the timing of microbiome sampling post-injury is crucial, as microbial populations can change rapidly in response to the physiological stress and interventions typical of acute SCI care. This variability was noted and highlighted the need for standardised methodologies in microbiome research to enhance comparability across studies (63). Additionally, many studies in this area suffer from small sample sizes, which can limit the statistical power of the findings and the conclusions that can be drawn. SCI populations are inherently diverse in terms of injury level, severity, and individual health conditions, which necessitates large, diverse study cohorts to capture the full scope of microbiome variations post-injury. The impact of small sample sizes is particularly significant in studies attempting to correlate specific microbiota changes with health outcomes, as the variability within small groups can overshadow subtle microbiome influences. This limitation emphasises the need for multicenter studies to gather adequate participant numbers that reflect the broad spectrum of SCI cases.

The complexity of accurately characterising the gut microbiome itself poses another significant hurdle. The microbiome is influenced by numerous factors, including diet, medication, pre-existing health conditions, and the environment, which all can confound results. Advanced techniques such as high-throughput sequencing and metagenomic analysis have provided deep insights into the microbial composition and its functional capabilities; however, these methods also require sophisticated data analysis tools and expertise. The complexity of data interpretation and the potential for overestimation of certain microbial functions or interaction points out the intricacies involved in decoding metagenomic data (64).

Finally, the ethical considerations in translating microbiome research into practical SCI therapies are non-trivial. Faecal microbiota transplantation and probiotic treatments raise ethical and safety concerns that must be rigorously addressed through controlled clinical trials and regulatory frameworks. The potential for adverse effects, such as infections from donor material in FMT or unanticipated impacts of probiotics on the gut ecosystem, must be balanced against the therapeutic benefits (65). Establishing causal relationships between microbiota alterations and outcomes in SCIs is a formidable challenge in microbiome research, primarily due to the multitude of confounding factors that can influence both the microbiome and SCI recovery. These factors include variations in diet, medication use, pre-existing health conditions, and different rehabilitation practices, all of which can significantly alter the gut microbial composition. For instance, antibiotics commonly used during SCI management can drastically reduce microbial diversity, complicating efforts to discern whether observed microbiota changes are a direct consequence of the SCIs or a result of the treatments administered. Additionally, dietary changes often recommended post-injury, such as increased fibre intake to manage bowel dysfunction, can independently affect the gut flora (66).

Moreover, the inherent delay between SCI onset and changes in the microbiota presents another challenge for researchers trying to establish causality. The microbiome's response to the altered physiological state post-injury may take weeks or even months to manifest, during which time many other variables are also changing. This delay complicates the ability to link specific microbial changes directly to injury mechanisms or outcomes (58).The variability in individual microbiomes adds another layer of complexity. Each person's gut microbiota is unique, influenced by genetics, early life exposures, lifestyle, and environmental factors. This individual variability means that even within similar groups of SCI patients, the microbiome's response to injury can differ dramatically, obscuring broad causal inferences (67, 68). Furthermore, the bidirectional nature of interactions between the microbiome and the host complicates the establishment of causality. The gut-brain axis implies that while the microbiome can affect brain and spinal cord function post-injury, the reverse is also true: the altered neurological state can influence gut functions and microbiota. This complex interplay challenges researchers to differentiate cause from effect in studies examining SCI outcomes and microbiota changes (69).

## 6 Future perspectives

The complexity and variability inherent in studying the gut microbiome's impact on SCI recovery underscore the urgent need for longitudinal studies with larger sample sizes. Long-term studies are crucial for understanding the dynamics of microbiota changes over time and their long-lasting effects on SCI outcomes. Larger cohorts would provide the statistical power necessary to discern subtle but clinically significant effects, reducing the influence of individual variability and enhancing the reliability of findings. For instance, large-scale longitudinal studies could help clarify the temporal relationship between microbiota alterations and SCI recovery, enabling researchers to identify potential windows for therapeutic intervention. Moreover, the establishment of standardised methodologies for microbiome analysis is essential to ensure consistency and comparability across studies. Currently, different studies employ various techniques for sampling, DNA extraction, and sequencing, which can lead to discrepancies in results. Standardising these methods would facilitate more accurate comparisons of data from different studies and improve the overall quality of research (70).

Controlled intervention trials are also pivotal in moving from observational to causal insights regarding the microbiome's effects on SCI recovery. By manipulating the microbiome through probiotics, prebiotics, dietary modifications, or faecal microbiota transplantation, and observing the outcomes, researchers can more directly assess the impact of specific microbial changes. Such trials would provide essential data on the efficacy and safety of microbiome-targeted therapies. Ultimately, integrating these approaches—longitudinal designs, standardised methodologies, and controlled trials—will significantly enhance our understanding of the microbiome's role in SCI recovery.

The potential for developing microbiome-based therapies as adjunct treatments for SCIs is gaining traction within the medical research community, driven by growing insights into the significant role the gut microbiome plays in overall health and disease management. Research indicates that the gut microbiome influences systemic inflammation, immune function, and even neuroprotection, which are all critical factors in the recovery process following SCI. Prebiotics and probiotics represent targeted interventions aimed at improving gut health through the modulation of the gut microbiome. Prebiotics, nondigestible food components such as inulin and fructo-oligosaccharides, serve as fuel for beneficial gut bacteria, promoting their growth and activity. Probiotics, on the other hand, are live microorganisms that, when administered in adequate amounts, confer a health benefit to the host. The consumption of probiotics has been associated with a range of health benefits, including improved digestive health, enhanced immune function, and a reduced risk of certain infections. Clinical trials and meta-analyses have supported the use of specific probiotic strains for the treatment and prevention of gastrointestinal disorders, underscoring the potential of probiotics' use as a therapeutic tool for managing secondary complications post SCIs (71, 72). On the other hand, FMT is a more direct approach to altering the gut microbiome, involving the transfer of stool from a healthy donor to a recipient with a dysbiotic gut microbiome. FMT has been primarily used in the treatment of recurrent Clostridium difficile infection, with a high success rate. The procedure has also been explored as a potential treatment for other conditions associated with gut microbiome dysbiosis, including inflammatory bowel disease, irritable bowel syndrome, and metabolic syndrome. While the mechanisms by which FMT exerts its effects are not fully understood, it is believed to restore microbial diversity and functionality, thereby re-establishing a healthy gut ecosystem. Ongoing research and clinical trials continue to investigate the safety, efficacy, and long-term outcomes of FMT for various other indications (36, 73).

Dietary interventions represent a more accessible method of influencing the gut microbiome. High-fibre diets, rich in prebiotics, can stimulate the growth of beneficial bacterial species that produce SCFAs, known for their anti-inflammatory properties. These dietary modifications can potentially reduce the chronic inflammation associated with SCIs and support neurological health. Personalised nutrition plans tailored to the needs of SCI patients could thus play a pivotal role in adjunct treatments, leveraging diet as a tool for microbiome management to enhance recovery outcomes (28).

Probiotics and prebiotics have emerged as promising modulators of gut microbiota, with potential implications for SCI recovery. Probiotics are defined as live microorganisms that, when administered in adequate amounts, confer health benefits to the host. Prebiotics, on the other hand, are non-digestible food components that selectively stimulate the growth and activity of beneficial gut bacteria. In the context of SCI, gut dysbiosis—characterised by a reduction in beneficial commensals and an increase in opportunistic pathogens—can exacerbate systemic inflammation and impair neuroimmune communication. Probiotic supplementation has been shown to restore microbial balance, enhance intestinal barrier integrity, and reduce pro-inflammatory cytokine levels. For example, strains such as *Lactobacillus rhamnosus* and *Bifidobacterium longum* have demonstrated anti-inflammatory effects and neuroprotective properties in preclinical models (74, 75). Prebiotics such as inulin, fructooligosaccharides, and galactooligosaccharides promote the growth of SCFA-producing bacteria, including *Faecalibacterium prausnitzii* and *Akkermansia muciniphila* (76, 77). SCFAs like acetate, propionate, and butyrate play a crucial role in modulating immune responses, maintaining epithelial integrity, and influencing central nervous system signalling via the gut-brain axis. Together, probiotics and prebiotics may offer a non-invasive strategy to mitigate gut dysbiosis and promote recovery following SCI. Future clinical studies are warranted to validate their efficacy and optimise strain-specific interventions tailored to individual microbiome profiles.

The integration of microbiome-based therapies with conventional SCI treatments presents a comprehensive approach that could synergise with existing pharmacological and rehabilitative strategies. For example, combining anti-inflammatory drugs with probiotics may enhance the overall therapeutic effects by simultaneously reducing inflammation and supporting gut health. Such integrative treatment plans would address multiple facets of SCI recovery, from neurological repair to immune system support, showcasing the holistic potential of incorporating microbiome-focused strategies into traditional care paradigms. The rising field of gut microbiota research offers promising new avenues for enhancing the treatment and recovery outcomes of SCIs. Insights into the gut-brain axis, the complex communication network linking the gastrointestinal tract and the central nervous system, suggest that the gut microbiota can significantly influence systemic inflammation, immune response, and even neuroplasticity. By manipulating this microbiota through targeted interventions like probiotics, prebiotics, or faecal microbiota transplantation, it may be possible to mitigate some of the secondary complications associated with SCIs, such as chronic inflammation and susceptibility to infections. Such strategies could complement existing therapeutic approaches, potentially accelerating recovery and improving overall patient outcomes. The interdisciplinary nature of this research underscores the necessity of integrating expertise from neurology, microbiology, nutrition, and rehabilitation therapy to develop effective microbiome-based treatments. Collaboration across these diverse fields can drive innovation by combining methodologies and insights, leading to a more comprehensive understanding of how the gut microbiome impacts SCI recovery. For example, neurologists and microbiologists can work together to map the mechanisms by which gut bacteria influence neuroinflammation, while nutritionists and rehabilitation therapists can develop dietary and exercise plans that support beneficial microbiome changes. This interdisciplinary approach not only enhances the depth of research but also ensures that therapeutic developments are holistic and grounded in a robust understanding of the interconnected systems affecting SCI patients. To advance the understanding of gut microbiome involvement in SCI, future research should prioritise dynamic, longitudinal studies that capture microbial shifts over time. Such studies can elucidate the temporal evolution of dysbiosis, identify critical windows for intervention, and correlate microbial changes with clinical outcomes. Integrating multi-omics approaches, including metagenomics, metabolomics, and transcriptomics with neuroimaging and immune profiling, will enable a systems-level view of host-microbiota interactions. Additionally, personalised microbial therapies tailored to individual microbiome signatures may offer targeted strategies for enhancing neuroprotection and functional recovery. Establishing standardised protocols for sample collection, sequencing, and data analysis will be essential to ensure reproducibility and clinical relevance.

## 7 Conclusion

The gut microbiome plays a pivotal role in shaping immune responses, maintaining intestinal integrity, and influencing neuroregeneration following SCI. SCI disrupts gut motility and barrier function, leading to microbial dysbiosis that can aggravate systemic inflammation and hinder recovery. This review highlights the multifaceted relationship between gut microbiota and SCI, emphasising the importance of specific microbial species, microbial-derived metabolites, and environmental factors such as ageing, diet, stress, and antibiotic exposure. Probiotics and prebiotics emerge as promising interventions to restore microbial balance and support neuroimmune modulation. Dietary patterns that promote short-chain fatty acid production may further enhance neuroprotection and functional outcomes. While current evidence is compelling, most findings are derived from preclinical models, and human studies remain limited. Future research should focus on dynamic, longitudinal profiling of the gut microbiome in SCI patients, integrating microbial data with clinical and neurological outcomes. Personalised microbial therapies, informed by individual microbiome signatures, could revolutionise SCI management and rehabilitation. Additionally, understanding the temporal evolution of microbial changes post-injury may help identify critical windows for intervention. In conclusion, the gut microbiome represents a novel and modifiable target in SCI recovery, and its integration into clinical practice holds promise for improving patient outcomes. Continued interdisciplinary research is essential to translate these insights into effective, evidence-based therapies.
